# Correction to “Impact of Growth Environment on the Crystal Growth and Magnetic and Electronic Properties of Ba_2_NiWO_6_ Single Crystals”

**DOI:** 10.1021/acs.cgd.5c01392

**Published:** 2025-10-18

**Authors:** Abanoub R. N. Hanna, A. T. M. Nazmul Islam, Daniel Abou-Ras, Clemens Ritter, Igal Levine, Ralf Feyerherm, Bella Lake

In the above-mentioned article, the name of coauthor Clemens Ritter was misspelled as “Clemes Ritter.” This notice corrects the spelling of Clemens Ritter in the byline and in the Author Information section. The online version and associated indexing/metadata should be updated accordingly.

